# Prevalence, determinants, and barriers to follow-up colonoscopy after a positive fecal immunochemical test in Qaemshahr, Iran: A population-based cross-sectional study from 2022 to 2023

**DOI:** 10.1016/j.pmedr.2025.103331

**Published:** 2025-12-01

**Authors:** Maryam Zarrinkamar, Mahbobeh Ebrahimi, Mahnaz Zahedi, Farzaneh Amini, Mojgan Geran, Erfan Ghadirzadeh

**Affiliations:** a Assistant Professor of Family Medicine, Department of Family Medicine, Faculty of Medicine, Mazandaran University of Medical Sciences, Sari, Iran; b Diabetes Research Center, Institute of Herbal Medicines and Metabolic Disorders, Mazandaran University of Medical Sciences, Sari, Iran; c Assistant Professor of Gastroenterology and Hepatology, Gut and Liver Research Center, Non-Communicable Diseases Institute, Mazandaran University of Medical Sciences, Sari, Iran; d MD, MPH Candidate, Mazandaran University of Medical Sciences, Sari, Iran; e Department of Biostatistics and Epidemiology, Student Research Committee, Faculty of Health, Mazandaran University of Medical Sciences, Sari, Iran; f Gastrointestinal Cancer Research Center, Non-Communicable Diseases Institute, Mazandaran University of Medical Sciences, Sari, Iran

**Keywords:** Colorectal neoplasms, Colonoscopy, Fecal immunochemical test, Screening

## Abstract

**Objective:**

Fecal Immunochemical Test (FIT) screening reduces mortality by detecting early-stage colorectal cancer (CRC), but its success depends on follow-up colonoscopy after a positive result. Follow-up rates vary widely, influenced by several barriers, which need to be addressed. Thus, the present study aimed to assess the prevalence, determinants, and barriers to a follow-up colonoscopy after a positive FIT result.

**Methods:**

This cross-sectional study evaluated FIT-based CRC screening from April 2022 to March 2023 in Qaemshahr, Iran. Positive FIT results triggered follow-up colonoscopies. Data on colonoscopy results and reasons for non-compliance were collected. Logistic regression was conducted to identify factors affecting follow-up rates.

**Results:**

Of 14,784 screened, 294 (1.98 %) had positive FIT results; only 167 (56.80 %) underwent colonoscopy, with 73.65 % showing no abnormalities. Barriers included cost (18.89 %), perceived unimportance (41.73 %), fear (34.64 %), and lack of social support and appointment difficulties (4.72 %). Lower education levels (OR from 15.46 for illiterate to 3.79 for high school level, compared to university/college level), and asymptomatic status (OR: 1.86; 95 %CI: 1.01, 3.41) significantly reduced follow-up odds; while other demographics showed no association.

**Conclusion:**

This study reveals a concerning 56.80 % follow-up colonoscopy rate among FIT-positive patients, driven by cost, communication gaps, fear, and educational disparities.

## Highlights

• Follow-up colonoscopy rate after positive fecal immunochemical test was only 56.8 %.
• Main barriers: cost, poor communication, fear, and logistical issues.
• Lower education level strongly reduced odds of follow-up colonoscopy.
• Asymptomatic patients were less likely to undergo follow-up colonoscopy.
• A dose-responsive trend was observed between education and follow-up.

## 1 Background

Screening programs have proven effective in reducing colorectal cancer (CRC) mortality by facilitating the early detection and removal of precancerous lesions or the identification of cancer at a stage when it is treatable (Hewitson et al., 2008). Among screening modalities, the fecal immunochemical test (FIT) has emerged as a cornerstone due to its non-invasive nature and cost-effectiveness (Lee et al., 2014).

Colonoscopy remains the gold standard for confirming these findings, as it allows for direct visualization and, if necessary, the removal of precancerous lesions (Rex et al., 2017). However, the success of FIT-based screening depends on patients completing the follow-up colonoscopy. Failure to do so undermines the screening's preventive potential, allowing undetected lesions to progress and potentially develop into more severe conditions. Studies have shown that individuals who do not undergo follow-up colonoscopy after a positive FIT are at significantly higher risk of CRC-related death (Zorzi et al., 2022).

Despite its importance, the prevalence of follow-up colonoscopy after a positive FIT varies widely across studies, from over 79 % in some European programs (Zorzi et al., 2020) to under 40 % in safety-net healthcare systems (Issaka et al., 2022) and approximately 30 % within a Medicaid health plan in the United States (Green et al., 2020). These disparities underscore a critical gap in screening efficacy, which is influenced by contextual factors such as the healthcare system's structure, patient demographics, and access to diagnostic services.

The determinants of follow-up colonoscopy are multifaceted, spanning patient-level factors (socioeconomic status, education, fear (Jones et al., 2010), and health literacy (Cusumano et al., 2020)) (Issaka et al., 2021), healthcare system-level barriers (ling wait times (Zorzi et al., 2020), costs, and insurance coverage), and provider-related issues (poor care coordination).

Low follow-up colonoscopy rates after a positive FIT test undermine the entire value of colorectal cancer screening. This leaves high-risk individuals vulnerable and wastes resources. Studying this problem is crucial to identify barriers, inform effective policies, and ensure screening actually prevents cancer. Thus, the present study aimed to assess the prevalence, determinants, and barriers to a follow-up colonoscopy after a positive FIT test.

## 2 Materials and methods

### 2.1 Study design and population

The present cross-sectional study was conducted in urban and rural health centers situated in Ghaemshahr, Iran, utilizing samples collected during April 2022 to March 2023. The study population consisted of individuals aged 50 to 70 years who underwent a FIT test as part of a CRC screening program. The program systematically targets all individuals aged 50 to 70 years, who are covered by the extensive network of urban and rural health centers across Qaemshahr, Mazandaran, Iran. The screening protocol utilizes a single round of the quantitative FIT test as the initial step. FIT kits were distributed and explained to eligible individuals by healthcare workers at the local health centers. This study has been approved by the Ethics Committee of Mazandaran University of Medical Sciences (IR.MAZUMS.IMAMHOSPITAL.REC.1402.124). Written informed consent was obtained from all participants prior to their participation in the study.

A census sampling strategy was adopted, encompassing all individuals who satisfied the inclusion criteria of performing a FIT test and attended urban and/or rural health centers in Ghaemshahr. The inclusion criteria were as follows:

1. Individuals aged 50 to 70 years who had undergone a FIT screening test.
2. A positive FIT result documented in their health records.

The sole exclusion criterion was patient dissatisfaction with participation.

Out of 14,784 participants screened with the FIT test, 294 (1.98 %) had positive results, with a mean age of 61.13 ± 5.31 years. A total of 160 (54.4 %) patients were female, 26 (8.8 %) patients had no formal education, 225 (76.5 %) patients had no symptoms, 11 (3.7 %) patients had a positive family history in their first degree relatives. In contrast, none of the patients had a positive family history in their second-degree relatives (Table 1).

**Table 1:** Baseline characteristics of the FIT positive patients in Qaemshahr, Iran, 2022 to 2023, the reason for not performing a follow-up colonoscopy, and colonoscopic findings in case if a follow-up colonoscopy was conducted. Data is presented in number (percent).

Variable		Total (n: 294)	Follow-up Colonoscopy		*P*-value
			Yes (n: 167)	No (n: 127)	
Age	50–55	34 (11.6)	16 (47.1)	18 (52.9)	0.15
	55–60	82 (27.9)	50 (61.0)	32 (39.0)	
	60–65	81 (27.6)	52 (64.2)	29 (35.8)	
	65–70	97 (33.0)	49 (50.0)	48 (49.5)	
Gender	Male	134 (45.6)	75 (56.0)	59 (44.0)	0.79
	Female	160 (54.4)	92 (57.5)	68 (42.5)	
Residence	Rural	236 (80.3)	134 (56.8)	102 (43.2)	0.98
	Urban	58 (19.7)	33 (56.9)	25 (43.1)	
Education	Illiterate	26 (8.8)	10 (38.5)	16 (61.5)	< 0.01
	Primary School	54 (18.4)	27 (50.0)	27 (50.0)	
	Secondary School	80 (27.2)	41 (51.2)	39 (48.8)	
	High School	109 (37.1)	68 (62.4)	41 (37.6)	
	College/University	25 (8.5)	21 (84.0)	4 (16.0)	
1st-degree family history	Yes	11 (3.7)	9 (81.8)	2 (18.2)	0.12
	No	283 (96.3)	158 (55.8)	125 (44.2)	
Symptoms	Yes	69 (23.5)	44 (63.8)	25 (36.2)	0.18
	Rectal Bleeding	13 (4.42)	11 (84.6)	2 (15.4)	
	Constipation	49 (16.66)	27 (55.1)	22 (44.9)	
	Weight Loss	5 (1.70)	1 (20.0)	4 (80.0)	
	Other	2 (0.68)	2 (100.0)	0 (0.0)	
	No	225 (76.5)	123 (54.7)	102 (45.3)	
Reason ^1^	High Cost	–	–	24 (18.9)	
	Unimportant ^2^	–	–	53 (41.7)	
	Fear ^3^	–	–	44 (34.6)	
	Systematic Issues ^4^	–	–	6 (4.7)	
Colonoscopy Findings	Polyp	–	23 (13.8)	–	
	Colorectal Cancer	–	8 (4.8)	–	
	Other ^5^	–	13 (7.8)	–	
	Normal	–	123 (73.6)	–	

^1^ Reason for not performing follow-up colonoscopy.

^2^ The significance of the procedure was not adequately communicated, or the patients perceived the provided explanations as neither essential nor critical.

^3^ Fear of procedure or its side effects.

^4^ Including the absence of a companion (social support) and difficulties in obtaining a doctor's appointment.

^5^ Including aphthous lesions (n: 1), hemorrhoids (n: 8), and ulcerative colitis (n: 4).

### 2.2 Measures

Data pertaining to FIT results were acquired through the following procedure:

1. Patients received a FIT kit along with detailed instructions during their visit to the health center.
2. Patients were directed to collect stool samples at home in accordance with the provided instructions.
3. Samples were returned to the health center for analysis.

Additionally, a physician at the health center assessed participants' family history of colorectal cancer and associated symptoms (including rectal bleeding in the past month, constipation in the past month, and significant weight loss [more than 10 % of body weight] in the past 6 months, etc.).

Healthcare professionals (typically a physician or a trained nurse) at the health centers contacted individuals with positive FIT results via telephone. A positive FIT result was defined as the detection of human hemoglobin (Hb) in a stool sample exceeding a pre-specified threshold of 6 μg/g (equivalent to 50 ng/mL). The structured follow-up process entailed the following steps, with a programmatic goal of completing the colonoscopy within 60 days of the positive FIT result:

First, patients were informed of their positive FIT result. The implications were explained in layperson's terms, emphasizing that the test detected hidden blood in the stool, which could be a sign of precancerous polyps or cancer, and that a colonoscopy was the necessary next step to find the cause. The critical role of colonoscopy in preventing cancer through the removal of polyps was stressed.

Second, patients were assisted in scheduling an appointment with a gastroenterologist for a diagnostic colonoscopy. The program aimed for this initial specialist contact to occur within 2–3 weeks of the positive result.

Third, following the referral, more detailed information was provided. This included a verbal explanation of the colonoscopy procedure itself, the importance of adequate bowel preparation for a successful examination, and the addressal of common fears regarding pain or potential complications. The conversation aimed to frame the colonoscopy as a potentially life-saving diagnostic step rather than just an optional procedure.

Then, primary healthcare staff made follow-up calls to patients who had been referred to monitor their progress, remind them of scheduled appointments, and identify any barriers (e.g., cost, transportation, fear).

Finally, after the colonoscopy was performed, the program relied on the gastroenterologist's standard practice to promptly report the findings and any histopathological results back to the patient and their primary health record.

This structured approach facilitated the systematic evaluation of positive FIT results and ensured an organized follow-up process for patients. However, the actual time to colonoscopy was subject to real-world constraints, including patient availability, gastroenterologist scheduling capacity, and the barriers detailed in the results.

All participants were subsequently contacted by telephone. For those who had undergone a colonoscopy, arrangements were made to obtain and record the results. In cases where a colonoscopy was not performed despite a positive FIT result, the reason was documented. For the purpose of this study, adherence to follow-up was defined as the completion of a colonoscopy within 180 days (6 months) of the positive FIT result.

### 2.3 Statistical analysis

Statistical analysis was performed using SPSS version 26 software. The analyses were categorized into descriptive and analytical components. Variables were reported as frequencies, percentages, mean, and standard deviation (SD). The chi-square test was used to compare variables between groups with and without follow-up colonoscopy, with a significance level set at 0.05. Subsequently, multiple logistic regression analysis was used to model the relationships between all covariate variables and the dependent variable (not performing a follow-up colonoscopy) to assess factors influencing the odds of not performing a follow-up colonoscopy after a positive FIT. The goodness-of-fit of the final model was assessed using the Hosmer-Lemeshow test and Nagelkerke R-square value.

## 3 Results

Out of 294 FIT-positive patients, only 167 (56.8 %) underwent a follow-up colonoscopy, with 123 (73.7 %) reporting no abnormal findings in the colonoscopy report. Reasons for not conducting a follow-up colonoscopy included high costs of the procedure (18.9 %), the significance of the procedure was not adequately communicated, or the patients perceived the provided explanations as neither essential nor critical (41.7 %), fear of procedure or its side effects (34.6 %), the absence of a companion (social support), and difficulties in obtaining a gastroenterologist's appointment for colonoscopy (4.7 %).

Additionally, multiple logistic regression analysis demonstrated that patients without symptoms (OR: 1.86; 95 %CI: 1.01, 3.41) and patients with lower levels of education (from OR: 15.46; 95 %CI: 3.27, 73.00 for illiterate patients to OR: 3.79; 95 %CI: 1.13, 12.62 for patients with high school level of education; compared to patients with university or college level of education) showed significantly higher odds of not performing a follow-up colonoscopy after a positive FIT (Table 2). However, gender, age, residential area, and family history showed no significant associations with performing a follow-up colonoscopy. Interestingly, the level of education could demonstrate a dose-responsive trend showing that patients with lower education levels are more likely not to perform a follow-up colonoscopy (OR: 3.79 for high school level, OR: 7.60 for secondary school level, OR: 9.28 for primary school level, and OR: 15.46 for illiterates).

**Table 2:** Multiple logistic regression results for determinants of not performing a follow-up colonoscopy after a positive FIT among FIT positive patients in Qaemshahr, Iran, 2022- to 2023.

Variables		OR	95 %CI
Age	50–55	1.85	0.75, 4.52
	55–60	0.98	0.48, 2.00
	60–65	0.61	0.32, 1.19
	65–70	Ref	Ref
Gender	Male	Ref	Ref
	Female	0.60	0.34, 1.06
Residence	Rural	1.13	0.59, 2.16
	Urban	Ref	Ref
Education	Illiterate	15.46	3.27, 73.00
	Primary school	9.28	2.36, 36.41
	Secondary school	7.60	2.19, 26.40
	High school	3.79	1.13, 12.62
	College/University	Ref	Ref
1st-degree family history	Yes	Ref	Ref
	No	3.64	0.69, 19.14
Symptoms	Yes	Ref	Ref
	No	1.86	1.01, 3.41

Nagelkerke R-square: 0.118, Hosmer and Lemeshow Test P-value: 0.803.

## 4 Discussion

The present study aimed to assess the prevalence, determinants, and barriers to a follow-up colonoscopy after a positive FIT test. This study provides valuable insights into the follow-up behaviors of patients who receive a positive FIT result, a widely used screening tool for CRC. Only 56.8 % patients underwent a follow-up colonoscopy, with reasons for non-compliance including high costs (18.9 %), inadequate communication about the procedure's significance (41.7 %), fear of the procedure or its side effects (34.6 %), and logistical challenges such as lack of a companion or difficulty securing an appointment (4.7 %). Logistic regression analysis revealed that asymptomatic patients and those with lower education levels were significantly less likely to pursue follow-up. Notably, a possible dose-responsive trend was observed with education level, while gender, age, residential area, and family history showed no significant associations.

To deepen the interpretation of these determinants, our findings can be effectively framed using the Health Belief Model (HBM). The HBM posits that health-related behavior is influenced by an individual's perception of their susceptibility to a condition, its severity, the benefits of an action, and the barriers to taking that action (Rosenstock, 1974). In our study, the dominant barriers of high cost, fear, and logistical challenges align directly with the HBM's ‘Perceived Barriers’ construct. The finding that asymptomatic patients were less likely to adhere (OR: 1.86) likely reflects a lower ‘Perceived Susceptibility’ to serious illness and a lower ‘Perceived Severity’ of the threat posed by a positive FIT result, as they lack tangible symptoms. Furthermore, the powerful dose-response relationship with education level strongly suggests that health literacy is a key underlying factor influencing all HBM constructs. Patients with lower education may struggle to understand the implications of a positive FIT (‘Perceived Susceptibility/Severity’), may not fully grasp the ‘Benefits’ of a colonoscopy in preventing cancer, and are likely less equipped to navigate the complex ‘Barriers’ of cost and logistics. The reason ‘inadequate communication of the procedure's significance’ reported by 41.7 % of non-adherent patients can be seen as a critical failure to provide an effective ‘Cue to Action’ and to adequately convey ‘Perceived Benefits’.

The follow-up rate of 56.8 % in this study is different than rates reported in other studies (79.8 % in Zorzi et al. (Zorzi et al., 2020), 62.1 % in May et al. (May et al., 2019), 57.7 % in Martin et al. (Martin et al., 2017), 43.3 % in Jakubauskas et al. (Jakubauskas et al., 2025), 40.8 % in Issaka et al. (Issaka et al., 2022), 35.9 % and 32.8 % in Green et al. (Green et al., 2020), and 31.1 % in Cusumano et al. (Cusumano et al., 2021)) which may suggest a gap in the CRC screening pathway, potentially due to healthcare system differences, patient demographics, or suboptimal follow-up protocols.

This study identifies high costs, inadequate communication, fear, and logistical issues as key barriers to follow-up. These align with previous findings. Green et al. (Green et al., 2017) reported that avoidance (including inattention in patients), logistical concerns about handling stool, and fear deterred completion of colonoscopies. Similarly, Jetelina et al. (Jetelina et al., 2019) demonstrated that patients reported costs and insurance-related challenges, logistical transportation difficulties, a lack of social support, concerns and fears about the FIT/colonoscopy process and its adverse effects, and poor health literacy as significant reasons for not undergoing a follow-up colonoscopy.

While these were reported by patients, they often point to underlying health system deficiencies. The barrier of ‘inadequate communication’ is not merely a patient perception but indicative of a systemic failure in patient education and provider communication skills. Effective communication is needed to translate a positive screening result into motivated action. Furthermore, the ‘systematic issues’ barrier, encompassing the absence of social support and difficulties in obtaining specialist appointments, highlights critical gaps in care coordination and navigational support. The lack of a structured patient navigation system to guide individuals through the complex pathway from a positive FIT to a completed colonoscopy is a significant system-level shortfall. Our findings suggest that interventions must target these multi-level factors simultaneously, from improving patient education and counseling (addressing HBM constructs) to implementing system-level solutions like patient navigation, streamlined referral processes, and financial support programs to mitigate cost barriers.

Similarly, Azulay et al. (Azulay et al., 2021) reported a lack of comprehension of the procedure's importance among patients as the most significant factor influencing follow-up colonoscopy performance. Additionally, the results indicated that older patients and patients with low socioeconomic status (cost issues) demonstrate lower adherence rates to follow-up colonoscopy. On the other hand, Kerrison et al. (Kerrison et al., 2022) showed that fear (specifically, fear of pain and discomfort during the procedure) and social support (from friends and family members) were among the key factors associated with performing a follow-up colonoscopy. Unlike our findings, Cusumano et al. (Cusumano et al., 2021) found that males and younger patients were more likely to undergo a follow-up colonoscopy; however, the present study found no association between age and gender and follow-up colonoscopy performance.

Additionally, as demonstrated by Issaka et al. (Issaka et al., 2021), the main barriers to colonoscopy completion included social determinants (such as language barriers and homelessness, no accompanying sociat supporters, and no access to restrooms for bowel preparation), organizational factors (including poor care coordination between primary-care and specialized-care clinics, staffing shortages, referral delays, insurance approvals, and employee turnover), and cognitive barriers (involving difficulties with proper bowel preparation process, low health literacy, fear of the procedure or the diagnosis, and inability to understand the importance of follow-up after an abnormal FIT result). The consistency across studies highlights the universal nature of these barriers, suggesting that interventions must address both patient perceptions and systemic issues to be effective.

The strong association between lower education levels and reduced likelihood of follow-up colonoscopy is a critical finding. The odds ratios demonstrate a possible dose-responsive trend: illiterate patients (OR: 15.46) were far less likely to follow up compared to those with a university education, with intermediate risks for primary (OR: 9.28), secondary (OR: 7.60), and high school (OR: 3.79) education levels. This mirrors findings by prior studies, which noted that higher literacy increased colonoscopy completion rates (Issaka et al., 2021; Jetelina et al., 2019). The trend likely reflects differences in health literacy, with more educated patients better equipped to understand the implications of a positive FIT and the need for follow-up. Additionally, asymptomatic patients were less likely to undergo colonoscopy (OR: 1.86), which could be explained by the fact that symptomatic patients are more motivated to pursue follow-up, while asymptomatic individuals may not perceive an urgent need for colonoscopy, underestimating the risk indicated by a positive FIT.

### 4.1 Clinical and health policy implications

Providers should use clear, concise explanations and visual aids to emphasize the importance of follow-up, even for asymptomatic patients. Tailored counseling could bridge comprehension gaps, particularly for individuals with lower educational backgrounds. High costs suggest a need for policy interventions, such as subsidizing colonoscopies for patients with a positive FIT result. Fear of the procedure indicates a need for educational campaigns to demystify colonoscopy, highlighting its safety. Asymptomatic patients require explicit messaging about the risks associated with a positive FIT, regardless of not having symptoms. Telemedicine follow-ups or mobile clinics could enhance access.

Also, based on a systematic review by Issaka et al. (Issaka et al., 2019), the following interventions were evaluated to improve CRC screening by FIT test, which health policy makers can implement to increase screening rates according to evidence-based medicine policies:

1. Directly mailing FIT kits to patients was the most effective with 21.5 % improvement in screening rates.
2. Offering FIT kits during flu vaccination significantly increased screening, with 15.9 % improvement.
3. Pre-FIT patient reminders, including letters or calls before FIT distribution, modestly improved screening, with a median increase of 4.1 %.
4. Post-FIT reminders, including follow-up calls or letters, resulted in a slight median improvement of 3.1 %.
5. Electronic reminders to providers to discuss FIT screening showed minimal or mixed effects, with one study reporting a 4.2 % improvement and others showing no significant change.
6. Customized messages addressing screening barriers did not significantly improve FIT completion compared to standard outreach.
7. Low-literacy educational materials (e.g., booklets, DVDs) showed no significant benefit over standard brochures.
8. Offering $5–$10 incentives for FIT completion did not significantly increase screening rates.

### 4.2 Limitations

Our investigation was limited by the lack of detailed socioeconomic data (such as specific income levels and type of health insurance) and direct psychological measures (such as validated scales for health literacy or perceptions of cancer risk and benefits of screening based on theoretical models like the HBM). The absence of this data prevents a more granular analysis of how economic status and specific psychological constructs interact with the identified barriers. Future prospective studies should collect comprehensive data on socioeconomic and psychological factors to provide a more holistic understanding of the determinants of follow-up behavior. Furthermore, while we assessed colonoscopy completion within a 6-month window, we did not analyze the precise time-to-colonoscopy for each patient.

## 5 Conclusion

This study reveals a concerning 56.8 % follow-up colonoscopy rate among FIT-positive patients in Iran, driven by cost, communication gaps, fear, and education disparities. While consistent with prior research on barriers and determinants, discrepancies with studies on age, gender, and residence highlight contextual differences. Addressing these findings requires a multifaceted approach: enhancing patient education, reducing financial and logistical barriers, and targeting vulnerable groups. Such efforts could enhance the efficacy of CRC screening, leading to earlier detection and a reduction in mortality. However, our study also highlights the need for future research to include detailed socioeconomic and psychological data to fully elucidate these complex determinants.

## Declaration of AI use

The authors acknowledge the use of DeepSeek AI (https://www.deepseek.com/) to improve the readability and grammar of this manuscript. This AI tool was used exclusively for language polishing and did not contribute to the conceptual development, analysis, or interpretation of the research data. The author has thoroughly reviewed and revised the AI-edited text and assumes complete responsibility for the final version of the work.

## CRediT authorship contribution statement

**Maryam Zarrinkamar:** Writing – review & editing, Validation, Data curation, Conceptualization. **Mahbobeh Ebrahimi:** Writing – review & editing, Supervision, Resources, Project administration, Data curation, Conceptualization. **Mahnaz Zahedi:** Writing – review & editing, Project administration, Data curation. **Farzaneh Amini:** Writing – review & editing, Software, Formal analysis, Data curation. **Mojgan Geran:** Writing – review & editing, Supervision, Methodology, Data curation, Conceptualization. **Erfan Ghadirzadeh:** Writing – review & editing, Writing – original draft, Visualization, Software, Methodology, Formal analysis, Data curation.

## Consent for publication

Not applicable.

## Ethics approval and consent to participate

This study has been approved by the Ethics Committee of Mazandaran University of Medical Sciences (IR.MAZUMS.IMAMHOSPITAL.REC.1402.124). Written informed consent was obtained from all participants prior to their participation in the study. All procedures performed in this study were in accordance with the ethical standards of the Institutional Research Ethics Committee of Mazandaran University of Medical Sciences and with the 1964 Helsinki Declaration and its later amendments or comparable ethical standards.

## Funding

This study was conducted with partial financial support from 10.13039/501100004160Mazandaran University of Medical Sciences (Grant No. 18982).

## Declaration of competing interest

The authors declare that they have no known competing financial interests or personal relationships that could have appeared to influence the work reported in this paper.

## Data Availability

The data are available upon reasonable request from the corresponding author.
